# Management of tuberculosis risk, screening and preventive therapy in patients with chronic autoimmune arthritis undergoing biotechnological and targeted immunosuppressive agents

**DOI:** 10.3389/fimmu.2025.1494283

**Published:** 2025-02-03

**Authors:** Andrea Picchianti-Diamanti, Alessandra Aiello, Chiara De Lorenzo, Giovanni Battista Migliori, Delia Goletti

**Affiliations:** ^1^ Department of Clinical and Molecular Medicine, “Sapienza” University, S. Andrea University Hospital, Rome, Italy; ^2^ Translational Research Unit, National Institute for Infectious Diseases “Lazzaro Spallanzani”- Istituto di Ricovero e Cura a Carattere Scientifico (IRCCS), Rome, Italy; ^3^ Istituti Clinici Scientifici Maugeri, Istituto di Ricovero e Cura a Carattere Scientifico (IRCCS), Tradate, Italy

**Keywords:** tuberculosis disease, TB infection, rheumatoid arthritis, psoriatic arthritis, ankylosing spondylitis, biologic DMARDs, JAK inhibitors, preventive therapy

## Abstract

Tuberculosis (TB) is the leading cause of death in the world from an infectious disease. Its etiologic agent, the *Mycobacterium tuberculosis* (Mtb), is a slow-growing bacterium that has coexisted in humans for thousands of years. According to the World Health Organization, 10.6 million new cases of TB and over 1 million deaths were reported in 2022. It is widely recognized that patients affected by chronic autoimmune arthritis such as rheumatoid arthritis (RA), psoriatic arthritis (PsA), and ankylosing spondylitis (AS) have an increased incidence rate of TB disease compared to the general population. As conceivable, the risk is associated with age ≥65 years and is higher in endemic regions, but immunosuppressive therapy plays a pivotal role. Several systematic reviews have analysed the impact of anti-TNF-α agents on the risk of TB in patients with chronic autoimmune arthritis, as well as for other biologic disease-modifying immunosuppressive anti-rheumatic drugs (bDMARDs) such as rituximab, abatacept, tocilizumab, ustekinumab, and secukinumab. However, the data are less robust compared to those available with TNF-α inhibitors. Conversely, data on anti-IL23 agents and JAK inhibitors (JAK-i), which have been more recently introduced for the treatment of RA and PsA/AS, are limited. TB screening and preventive therapy are recommended in Mtb-infected patients undergoing bDMARDs and targeted synthetic (ts)DMARDs. In this review, we evaluate the current evidence from randomized clinical trials, long-term extension studies, and real-life studies regarding the risk of TB in patients with RA, PsA, and AS treated with bDMARDs and tsDMARDs. According to the current evidence, TNF-α inhibitors carry the greatest risk of TB progression among bDMARDs and tsDMARDs, such as JAK inhibitors and anti-IL-6R agents. The management of TB screening and the updated preventive therapy are reported.

## 1 Introduction

Tuberculosis (TB) is the leading cause of death in the world from an infectious disease. Its etiologic agent, the *Mycobacterium tuberculosis* (Mtb), is a slow-growing bacterium that has coexisted in humans for thousands of years. According to the World Health Organization (WHO), 10.6 million new cases of TB and over 1 million deaths were reported in 2022 (1).

As a respiratory pathogen, the transmission occurs through inhalation of aerosols or droplets containing bacilli expelled by a person with TB disease. An estimated quarter of the world population has been infected with Mtb (1). Most TB cases are reported in low- and middle-income countries. In particular, more than two-thirds of people with TB live in Bangladesh, China, India, Indonesia, Nigeria, Pakistan, Philippines and South Africa (1). This heterogeneous distribution is due to the differences between countries in terms of social and economic development and health-related factors, such as alcohol use disorders, diabetes, HIV infection, smoking and undernourishment, which are known to increase the risk of TB disease (2). In addition to health conditions, immunosuppressive therapies affecting the immune system, including those used for rheumatoid arthritis (RA), psoriatic arthritis (PsA) and ankylosing spondylitis (AS), increase the risk of TB disease in Mtb-infected individuals (3).

Following infection, the majority (90%) control Mtb replication through innate and adaptive immunity establishing a state referred to as TB infection (TBI), and in the past called latent TB infection (4). On the other hand, 5-10% of the infected subjects can develop TB disease; half of them within the first 5 years, and half during their lifetime.

TB is traditionally classified as primary or secondary according to the time between the initial infection and the onset of the clinical disease. Primary TB occurs in previously uninfected subjects after *de novo* infection, whereas secondary TB develops in a previously sensitized host, and it may occur following reactivation of Mtb infection or reinfection from external source. Indeed, secondary TB usually, but not always, develops in a person with a weakened immune system (5).

In the context of Mtb infection, a dynamic equilibrium between the host and the microbe is present, with bacilli that can switch from a dormant state to intermittent or active replication depending on the capability of the host immune system to contain or not Mtb replication (6, 7). Therefore, TB is referred to as a “continuum process” characterized by different stages between TB infection and TB disease, as described elsewhere (8, 9).

In this review, we revised the current evidence from randomized clinical trials (RCTs), long-term extension studies (LTEs), and real-life studies regarding the risk of TB in patients with chronic autoimmune arthritis including rheumatoid arthritis (RA), psoriatic arthritis (PsA), and ankylosing spondylitis (AS), treated with biologic and targeted disease-modifying immunosuppressive anti-rheumatic drugs (bDMARDs; tsDMARDs).

## 2 Immunopathogenesis of TB

The immune response to Mtb infection is multifaceted and it involves both innate and adaptive immune response (4, 10). Upon infection, bacilli are phagocytosed by alveolar macrophages, which represent the first defense line against Mtb due to their antimicrobial mechanisms (11, 12). However, Mtb has evolved different mechanisms to avoid its elimination by inhibiting phagosome maturation and acidification, and escaping autophagy in macrophages (13–15), which become a permissive niche for Mtb replication.

As the infection progresses, macrophages migrate into the lung interstitium where they recruit other innate cells such as neutrophils, monocytes, macrophages, and dendritic cells due to the release of cytokines, including TNF-α, IL-1α, IL-6, IL-1β and IFN-γ, thus favouring the dissemination of mycobacteria to uninfected cells. Once activated, T and likely B cells are recruited to the site of infection contributing to the formation of the organized granuloma, a structure known as the hallmark of TB (10). The immune microenvironment within the granuloma influences the prognosis and outcome of TB disease leading to different scenarios: Mtb clearance, bacterial replication causing primary TB, bacterial dormancy, or reactivation of the infection (16–19).

CD4^+^ Th1 cells producing cytokines such as IFN-γ and TNF-α have been identified as the most important cell subset to control Mtb infection. The differentiation of naïve CD4^+^ T cells to Th1 cells is promoted by IL-12, a cytokine released by antigen presenting cells (APCs) (20). IFN-γ and TNF-α enhance the antibacterial activity of macrophages by increasing autophagy, promoting phagosome maturation, and inducing the production of antimicrobial peptides. Besides macrophages, IFN-γ and TNF-α activate B cells and the cytotoxic CD8^+^ T cells. Both cytokines are of utmost importance for the formation and maintenance of a well-organized granuloma (21).

The role of Th17 cells, whose differentiation is induced by IL-23, is controversial. Th17 response seems to be involved in the early steps of protection from Mtb infection, and the recruitment of neutrophils, macrophages, and Th1 cells to the site of infection (22, 23). Th17 cells enhance the expression of cytokines (IL-17A, IL-17F, IL-21 and IL-22) and antimicrobial peptides that lead to phagocytosis of Mtb (23). IL-17 may be released by either innate lymphocytes of the ILC3 class or Th1/Th17, and it seems to be implicated in the maturation process of granulomas (24). However, an overproduction of IL-17 was also associated with exaggerated recruitment of neutrophils and inflammation leading to immunopathology (25, 26). As with IL-17, also the excessive production of other pro-inflammatory cytokines such as TNF-α, IL-1, IFN-γ may result in tissue damage and bacterial growth. A balance is crucial to control progression to TB disease (27).

The pivotal role of IL-12, IFN-γ, and TNF-α in controlling Mtb infection is corroborated by the higher susceptibility to TB disease of the individuals treated with immunosuppressive therapies like TNF-α inhibitors (28–30), or individuals with innate defects of the IL-12/IFN-γ axis (31–33), with HIV infection (34) or with primary immunodeficiencies associated with T-cell deficiency (35).

Mendelian susceptibility to mycobacterial disease (MSMD) is an inborn error of immunity associated with a selective predisposition to mycobacterial infections. MSMD involves specific mutations in 18 genes (*IFNG, IFNGR1, IFNGR2, STAT1, IL12B, IL12RB1, IL12RB2, IL23R, RORC, TBX21, IRF8, SPPL2A, ISG15, TYK2, JAK1, ZNFX1, NEMO, CYBB*), which are associated with an impaired IFNγ/IL-12 response/production (35). Moreover, patients with defects of CD40 ligand (CD40L) and NF-kB signaling are more susceptible to mycobacterial disease, as this pathway is involved in the IL-12 production (36).

A number of distinct Mendelian disorders are also caused by inborn errors in components of the IL-6 family of cytokines and their signaling pathways (STAT3 and GP130) (37). The majority of patients with TYK2 defects, one of the three Janus kinases (JAKs) associated with GP130 signaling, shows defects in type I antiviral and mycobacterial immunity (38).

In addition to the use of TNF-α inhibitors, the inherited TNF deficiency has been identified as a genetic aetiology of recurrent pulmonary TB in adults observed within 1 year of the end of treatment. TNF deficiency seems to be responsible for the selective impairment of reactive oxygen species (ROS) production by alveolar macrophages. The ROS production is crucial for the phagocytic control of Mtb (39).

Regarding B cells and antibodies (Abs), initially there was some scepticism about their effective contribution to the host defense against Mtb due to the intracellular nature of the pathogen (40, 41). However, although B cells and Abs may not be able on their own to counteract Mtb, the accumulating evidence shows that they can favour and enhance cell-mediated immunity (42). Indeed, Abs binding to Mtb can mediate different processes such as antibody-dependent cellular cytotoxicity, antibody-dependent cellular phagocytosis, and complement activation, thus helping to reduce the mycobacterial burden (42). However, B cells are not limited to antibody production. They can act as antigen-presenting cells by presenting mycobacterial antigens to T cells, thereby inducing their activation. In addition, once activated, B cells can release cytokines, thus affecting the activity of different immune cells (43, 44). The role of B cells in controlling Mtb infection is corroborated by the association of TB disease with reduced B cell count and function (45, 46).

**Figure 1:**
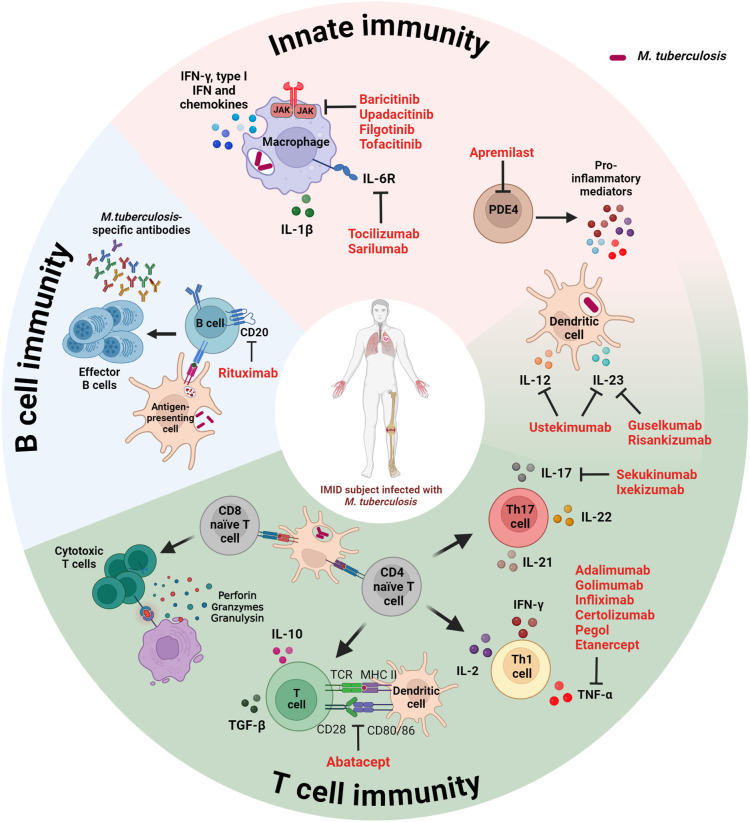
Schematic representation of the immune response targets of disease-modifying immunosuppressive anti-rheumatic drugs used for the management of IMID patients. Both innate and adaptive immunity (B and T cells) play a key role in controlling Mtb infection. Immunosuppressive therapies targeting host immune factors increase the risk for progression to TB. IMID, immune-mediated inflammatory disease; IFN, interferon; IL, interleukin; JAK, Janus kinase; MHC, major histocompatibility complex; PDE4, phosphodiesterase-4; TCR, T cell receptor; TGF, tumor growth factor. Created with BioRender.com.

Considering the pivotal role played by the host immune response in controlling Mtb replication, risk factors for the progression to TB disease include immunosuppressive therapies (**Figure 1**).

## 3 Patients with chronic autoimmune arthritis and TB risk

Patients with chronic autoimmune arthritis such as RA, PsA and AS are at higher risk of infections and related complications, which are the main cause of mortality in these conditions (47).

It is widely recognized that patients affected by RA, PsA, and AS have an increased incidence rate of TB disease compared to the general population, primarily related to immunosuppressive therapy rather than the disease itself. As conceivable, the risk is associated with age ≥65 years and is higher in endemic regions, but the use of immunosuppressive therapy plays a key role also in low TB endemic countries (3, 48). There is evidence that corticosteroids (CCS) can increase the risk of TBI reactivation in a dose-dependent manner (49, 50).

Among csDMARDs commonly used for the management of RA, PsA, and AS, leflunomide and azathioprine have emerged as having the highest risk, whereas sulfasalazine and methotrexate appear to confer only low to moderate risk (50, 51). In the last 20 years, biologic DMARDs (bDMARDs) have significantly improved the treatment of rheumatologic diseases, including RA, PsA, and AS, contributing to introduce the ambitious target of remission/minimal disease activity.

Anti-TNFα agents were the first bDMARDs to be adopted and are still the most used worldwide. Currently, 4 anti-TNF monoclonal antibodies and one receptor fusion protein are available: adalimumab (ADA), etanercept (ETN), infliximab (IFX), golimumab (GOL), and certolizumab (CTP). Other bDMARDs are used exclusively for the treatment of RA, including the anti-IL6 receptors tocilizumab and sarilumab, as well as the anti-CD20 rituximab. In contrast, the anti-IL12/23 ustekinumab, anti-IL23 guselkumab and risankizumab, and IL-17 inhibitors secukinumab and ixekizumab are used only for PsA and AS. Finally, the CTLA4-Ig abatacept and JAK inhibitors (JAK-i) are approved for both RA and PsA/AS. The increased risk of TB in patients with chronic autoimmune arthritis undergoing anti-TNF agents is widely recognized (3, 52–55). Although the data are less robust, there are also substantial data available for other bDMARDs such as rituximab, abatacept, tocilizumab, ustekinumab, and secukinumab (56–58).

Conversely, data on anti-IL23 agents and JAK inhibitors (JAK-i), which have been more recently introduced for the treatment of RA and PsA/AS, are scarcer.

## 4 Specific biological therapy for autoimmune arthritis and TB risk

In the following section, we describe the mechanism of action of bDMARDs and tsDMARDs used for the treatment of RA, PsA, and AS, and analyze how inhibiting specific pathogenic pathways might affect the integrity of the TB granuloma. We then summarize the main data on TB risk associated with these therapeutic agents from RCTs, LTE, and real-world studies in these pathologies (**Table 1**). Indeed, it is important to underline that RCTs provide very reliable and complete data, but patients with TB disease were not included, and patients with TBI could be enrolled only after having received TB preventive therapy according to local guidelines. On the other hand, real-life studies (registries and observational studies) are affected by a higher risk of bias, but can also report cases of TBI patients who did not receive preventive treatment; thus, they are particularly relevant for inferring the impact of b/tsDMARDs on the natural course of TBI.

**Table 1:** TB risk associated to the different biological drugs used for rheumatic patients.

Mechanism of Action	Biologic	Rheumatologic indications	TB risk	TB screening mandatory
TNF Inhibitors	Infliximab Adalimumab Etanercept Golimumab Certolizumab Pegol	RA, PsA, SpA	High High Medium/High Medium/High Medium/High	Yes
IL-6R Inhibitors	Tocilizumab Sarilumab	RA	Medium	Yes
JAK Inhibitors	Tofacitinib Baricitinib Upadacitinib Filgotinib	RA, PsA, SpA RA RA, PsA, SpA RA	Medium	Yes
CTLA4-Ig	Abatacept	RA, PsA	Low	Yes
IL-12/23 Inhibitor	Ustekinumab	PsA	Low	Yes
IL-23 Inhibitors	Guselkumab Risankizumab	PsA	Low	Yes
IL-17 Inhibitors	Secukinumab Ixekizumab	PsA, SpA	Low	Yes
CD20 Inhibitor	Rituximab	RA	Low	No
PDE4 Inhibitor	Apremilast	PsA	Low	No

*Risk based on mechanism of action and TB IR before the introduction of systematic TB screening.

### 4.1 TNF-α inhibitors

TNF-α is a pivotal cytokine in the pathogenesis of RA, PsA, and AS, as it acts on different cells such as synoviocytes, macrophages, chondrocytes, and osteoclasts. It induces local inflammation and pannus formation, contributing also to cartilage degradation and bone erosions (59, 60). High TNF-α levels have been observed in the synovial fluid and synovium of patients with RA and PsA (60).

As previously mentioned, TNF-α is also critical for the formation and maintenance of Mtb granulomas. TNF-α enhances the phagocytic capacity of macrophages, promotes the production of reactive nitrogen and oxygen species to kill intracellular bacteria, and facilitates the recruitment of immune cells at the site of infection​ (21).

TNF-α inhibitors neutralize TNF-α activity, by disrupting the immune response necessary for granuloma integrity, and leading to mycobacterial growth and dissemination with progression to TB disease.

There are remarkable differences among anti-TNF-α agents in their ability to inhibit TNF-α, that can explain the reported differences in TB risk. Anti-TNF-α mAbs target and neutralize both soluble and membrane-bound TNF-α with high affinity, and also have some cross-reactivity with Lymphotoxin (LT)-α (61). On the other hand, ETN, being a dimeric fusion protein, binds to the trimeric form of soluble TNF-α and, only to a lesser extent, to membrane-bound TNF-α and LT-α (62, 63).

The more comprehensive blockade of TNF-α activity and functions in immune defense mechanisms​ by anti-TNF-α mAbs may contribute to the observed higher risk of TBI reactivation.

The early clinical trials of IFX and ETN revealed a significant risk of TB, leading to the introduction of mandatory TB screening guidelines for patients starting anti-TNF-α therapy.

In particular, in the ATTRACT and ASPIRE RCTs there were 70 cases of TB disease among patients receiving IFX for an incidence rate (IR) of approximately 0.5-1.0/100 patient-years (PY); the majority of these cases occurred within the first few months of therapy (64).

ETN showed a lower incidence of TB in its pivotal ERA trial; however, the risk was still significant enough to warrant concern with an IR of 0.02-0.1/100PY (65). By the early 2000s, both the Food and Drug Administration (FDA) and the European Medicines Agency (EMA) had issued guidelines recommending TB screening before starting treatment with anti-TNF-α agents.

The subsequent implementation of screening protocols has significantly reduced TB rates, contributing to the lower incidence observed in LTE studies and real-world applications of IFX, ADA, and ETN, as well as with newer anti-TNF-α agents like golimumab and certolizumab pegol (52). Indeed, the IR of TB reactivation in clinical trials and LTEs conducted after the introduction of TB screening, decreased to 0.2-0.3 for IFX and to 0.1-0.2/100PY for ETN and ADA (66–79).

Also, golimumab and certolizumab pegol trials reported relatively low rates of TB. Indeed, the GO-BEFORE and GO-FORWARD golimumab studies found an IR of 0.2/100PY, whereas the RAPID 1-2 certolizumab trials found an IR of 0.1/100PY (80–84).

Different registries evaluated the incidence of TB in patients receiving TNF-α inhibitors after the introduction of TB screening protocols with similar results. Among these, the RABBIT registry reported an IR of 0.14/100PY, the BIOBADASER found an IR of 0.05-0.1/100PY, while the ARTIS registry reported an IR of 0.15/100PY (85–87).

Data from previous systematic reviews and meta-analyses showed similar data, with an IR of 0.18/100PY in rheumatic patients receiving anti-TNF-α, 4 times higher compared to rheumatic patients not receiving these therapies (88). Rigorous screening and prophylactic treatment are required on the summary of product characteristics (SmPC) of anti-TNF-α agents.

### 4.2 Anti-IL6R

Tocilizumab (TCZ) is a humanized monoclonal antibody targeting the human IL-6 receptor (IL6R). It was approved by EMA in 2009 for the treatment of RA patients. Sarilumab is another fully human monoclonal antibody targeting the IL-6R, more recently approved for RA treatment.

IL-6 is a versatile cytokine with a wide array of functions, including modulation of acute phase reactant pathways, B and T lymphocytes, blood-brain barrier permeability, synovial inflammation, and hematopoiesis. This cytokine plays a crucial role in bridging innate and adaptive immune responses, and in facilitating the recruitment of macrophages. Dysregulation of the IL-6 axis is implicated in the inflammatory pathways of various autoimmune disorders, such as RA.

Previous studies on experimental mice models showed that IL-6 plays a significant role in the protection against Mtb, and the absence of IL-6 leads to an early increase in bacterial load with a concurrent delay in the IFN-γ induction. However, IL6 knockout mice contained and controlled bacterial growth and developed a protective memory response to secondary infection, demonstrating that while IL-6 is involved in stimulating early IFN-γ production, it is not essential for the development of protective immunity against Mtb. The role of IL-6 in human TB remains controversial, and the specific functions of the IL-6 produced by B cells are still poorly understood, despite its abundance in TB-infected lungs. Some studies have reported increased concentrations of IL-6 in the sera of patients with advanced pulmonary TB compared to healthy controls, as well as elevated IL-6 gene expression in peripheral blood cells of TB patients, supporting a potential pathophysiological role (89, 90).

Furthermore, a recent study demonstrated that treatment with *in vitro* TCZ does not inhibit IFN-γ-specific response on whole blood from patients with TB disease stimulated with two different Mtb antigens, differently from the effects observed with ETN and IFX, both of which led to a reduced IFN-γ response (57, 91, 92).

A comprehensive safety analysis and systematic review published in 2014 assessed the incidence of TB in patients treated with TCZ from RCTs and LTE studies, and found no cases of TB disease among 15485 RA patients (29). A meta-analysis of RCTs trials and LTEs found 9 cases of TBI reactivation on 12509PY for an IR of 0.069/100PY (93). Data from the British Society for Rheumatology Biologics Register for Rheumatoid Arthritis (BSRBR-RA) showed one case of TB disease among 2171 RA patients treated with TCZ, resulting in an IR of 0.026/100PY (94).

Observational studies from European countries and a Japan nationwide study did not detect TB cases in TCZ users (95). Finally, a recently published nationwide observational study on RA patients from Korea, an intermediate TB burden country, reported 10 TB cases on 2185PY for an IR of 0.45/100PY (96).

The IR was similar to ETN and higher in TBI patients than those without TBI, indicating different effects between *de novo* infection and TB reactivation. Screening for TBI is mandatory in the SmPC of IL6R inhibitors.

### 4.3 Anti-IL-17

The IL-17 family encompasses six proteins (IL-17A to IL-17F) and five receptors (IL-17RA to IL-17RE). While IL-17A and IL-17F individually possess limited inflammatory potency, their robust inflammatory effects primarily stem from their ability to recruit immune cells and synergize with other pro-inflammatory cytokines like TNF-α, IL-1β, and IL-22. Through the recruitment and activation of neutrophils, IL-17A and IL-17F serve as pivotal components in the innate immune response against extracellular bacteria and fungi. Their protective role is particularly important on mucosal surfaces and skin, where they are rapidly released upon appropriate stimulation, thereby serving as a crucial link between innate and adaptive immune responses.

The involvement of IL-17 in TB pathogenesis has been debated, raising concerns and uncertainties about TB risk. Indeed, early granuloma formation may depend on IL-17A, but IL-17A-induced neutrophil recruitment may also increase pathological lesions and bacterial burden in chronic pulmonary infections. Notably, an *in vitro* study on a microgranuloma human model demonstrated that anti-TNF-α treatment could induce Mtb reactivation, whereas anti-IL17 treatment was comparable to control, indicating a lack of effect on Mtb dormancy. Moreover, mice lacking both IL-17RA and IL-22 pathways still managed to control TB, suggesting no compensatory relationship between these pathways. In contrast, TNF-α-deficient mice succumbed rapidly (97). In a series of studies by Khader and Cooper, low-dose aerosolized bacteria were delivered to the lower airways of the lungs in IL-17/IL-23 deficient mice (98, 99). Notably, the absence of IL-23 and IL-17 in the lung leads to more severe inflammation, suggesting that these cytokines help maintaining the granuloma integrity in later stages of Mtb-induced inflammation by limiting neutrophil death.

Secukinumab (SEK), a fully human monoclonal IgG1 antibody, specifically targets and inhibits IL-17A. Following the demonstration of its significant efficacy in phase 3 studies in 2015, it was approved for treating PsA and AS in 2016. Pooled data from 5 phase III trials on PsA (FUTURE program), and 4 phase III trials (MEASURE program) on AS, on a total of 2523 and 977 patients respectively, reported 5 cases of new TBI (one PsA and 2 AS patients), and no cases of TB disease (100). In LTE studies (1–5 years) in patients with PsA and AS, the safety profile was consistent with that of previous phase III studies, and no new TB infections or TB disease reactivations were observed (101). Notably, Liu et al. reported zero cases of reactivation among 3 PsA/AS patients with TBI who did not receive TB preventive therapy (102). Ngoc et al. in 2022 described a case of TB disease from Vietnam in a 19-years-old man affected by AS after two years of SEK treatment (103).

Ixekizumab (IXE) also neutralizes IL-17A but, differently from SEK, it is a humanized IgG4 monoclonal antibody. This structural variation contributes to its higher affinity for IL-17. Its approval for PsA and AS was granted in 2018. No TB disease cases from pooled analysis of 3 RCTs (SPIRIT program) on PsA patients were recorded. In the PsA studies, 32 (2.9%) patients resulted in TBI during the study, of whom 20 were discontinued per-protocol. Interestingly, among the remaining 12 patients continuing IXE treatment, no cases of TB reactivation were reported, even though only 7 patients received TBI therapy (104).

Finally, bimekizumab (BMK) represents a humanized IgG1 monoclonal antibody with dual neutralizing effects against both IL-17A and IL-17F. It has been very recently approved for the treatment of PsA and AS. A total of 267 PsA patients from BE COMPLETE trial and its open-label extension 1-yr follow-up (BE VITAL trial), reported no cases of TB disease (105). Data from two phase III BE MOBILES trials on AS, did not report TB disease cases at 1-yr follow-up. Finally, a total of 303 patients with active AS from BE AGILE trial and its open-label extension study reported no cases of TB disease (106, 107). We still need data from real-life studies and longer follow-ups. TB screening is only suggested in the SmPC of anti-IL17 agents.

### 4.4 Anti-IL-23 and IL-12/23

IL-12 and IL-23 are heterodimeric cytokines containing p35 and p40 subunits, and p19 and p40 subunits, respectively. IL-12 and IL-23 are produced by APCs, such as dendritic cells, macrophages, and monocytes. IL-12 plays a key role in the differentiation of naïve CD4^+^ T cells into Th1 cells, whereas IL-23 is involved in the expansion and maintenance of Th17 cells. A role for IL-12 and IL-23 dysregulation in the pathophysiology of PsA has been suggested (108).

Ustekinumab (UST) is a human monoclonal antibody that binds the p40 subunit shared by both IL-12 and IL-23, effectively suppressing their functions. It was approved for the treatment of PsA patients in 2013. No TB disease cases were observed neither in pivotal studies (PSUMMIT program) on a total of 1073 PsA patients, nor in their LTE data (109, 110). Few data from available real-life observational studies on PsA patients did not report cases of TB reactivation, and previous reviews have assessed the risk of TB reactivation as very low (111). A case of peritoneal TB in a PsA patient from Philippines, on UST treatment, with multi bio-failure, and after having been treated for latent TB, was observed (112). Screening and treatment of TBI are recommended in the SmPC supplied with UST.

Guselkumab (GUS) is a fully human IgG1λ monoclonal antibody, which specifically binds to the p19 subunit of IL-23. It stands as the first of its class to gain approval for the treatment of PsA patients.

Risankizumab (RSK) is a humanized immunoglobulin G1 monoclonal antibody that specifically inhibits IL-23 by binding to its p19 subunit, and it has been recently approved to treat PsA. Pooled data from DISCOVER 1 and 2 trials on 748 patients with active PsA for GUS reported zero cases of TB reactivation at 1-year follow-up (113, 114). RSK safety data sets from 4 phase II and III trials (KEEPsAKE program) in PsA on a total 1542 patients representing 2741.6PY, reported one case of TB disease in a patient from Taiwan previously treated with a 9-month course of isoniazid prophylaxis (115, 116). There is still very little real-life data on anti-IL-23 in PsA patients with RSK. Takeda et al. reported the case of a 64-year-old man affected by PsA who developed active pulmonary TB after two months of GUS therapy (the patient was negative at baseline TB screening) (117).

Notably, several real-world data are available for patients with psoriasis. A total of 68 and 25 TBI patients, who did not receive any or adequate TBI preventive therapy, were treated with RSK and GUS, respectively (118–122). Remarkably, there were no documented cases of TB reactivation, which corroborates the safety profile of anti-IL-23 agents in patients with TBI who did not receive prophylactic care. In the SmPC of anti-IL-23 agents is indicated that patients should be evaluated for TBI before starting treatment.

### 4.5 Anti-CD20

Rituximab (RTX) is a chimeric monoclonal antibody targeted against CD20, which is expressed on the surface of normal and malignant B lymphocytes. It was first approved by the FDA in 1997 for the treatment of malignancy, and in February 2006 for the treatment of patients with moderately to severely active RA, who did not adequately respond to one or more anti-TNF-α agents. RTX binds via its F(ab0)2 portion to the CD20 antigen expressed on B lymphocytes, whereas its Fc domain plays immune effector functions to mediate B cell lysis *in vitro*. RTX cytotoxicity is mediated by three different mechanisms including antibody-dependent cellular cytotoxicity, complement-dependent cytotoxicity, direct disruption of signaling pathways, and triggering of apoptosis (123, 124).

RTX’s targeted action on B cells, which spares the critical TNF-α pathways necessary for TB containment, likely explains its lower associated risk of TB reactivation compared to anti-TNF-α therapies as reported by several data (125–127). No cases of TB disease have been reported in patients receiving RTX in 9 RCTs conducted in 3623 RA patients. In two LTEs in RA patients, two cases of TB disease have been reported during a follow-up time of 9.5 years (IR 0.018/100PY) (29, 128). Data from real-life studies and registries found very few cases of *de novo* TB infection or TB disease reactivation during RTX treatment (129–132).

Data from observational studies and registries reported very few cases of TB disease. In particular, only one TB case was reported in 2484 RA patients treated with RTX in the German GENIRIS study, and 2 cases from the BSRBR-RA registry during 17154PY (0.012/100PY) (127, 133).

Finally, a meta-analysis including data from several clinical trials and LTEs reported that the IR of TB disease was high (>0.040/100PY) for patients treated with tofacitinib and all biologics but RTX (0.020/100PY) (93). Overall RTX emerged as having one of the lowest pooled IR of TB among bDMARDs. In line with this evidence, the summary of product characteristics of RTX does not specifically mandate routine screening for TB before initiating therapy.

### 4.6 CTLA-Ig

Abatacept (ABT) is a fully human recombinant fusion protein composed of the extracellular binding domain of human cytotoxic T lymphocyte-associated antigen-4 (CTLA-4), fused to a modified segment of human IgG1. Its mechanism of action involves blocking the CD80/CD86 costimulatory pathways, thus preventing the activation of naïve T cells. ABT binds to CD80 and CD86 on APCs with higher affinity compared to CD28 on T cells, effectively interfering with the interaction between CD28 and CD80/CD86 (134). ABT has been approved by the EMA for the treatment of RA since 2007, 10 years later it received approval for the treatment of PsA patients.

Its peculiar mechanism of immune modulation, which avoids direct cytokine inhibition, seems to be a key factor that likely contributes to its safer profile compared to anti-TNF-α regarding the overall risk of infections and, in particular, TB reactivation.

Pooled data from 8 RCTs and LTE studies on RA patients revealed an overall IR of 0.0066/100PY (135). A recent 10-year international post-marketing study found a very low IR across different registries (ARTIS, FORWARD, RABBIT) with only one event on a total of 9652PY of exposure (136). TB rates were low but slightly higher in two Canadian and USA registries, with 9 cases on 1067PY and 17 cases on 3994PY, respectively (137). Finally, a recent meta-analysis of LTEs showed a low estimated pooled IR of 0.07/100PY (93). Despite these reassuring data, screening for TBI is recommended in the SmPC of abatacept.

### 4.7 Apremilast

Apremilast, an oral phosphodiesterase-4 (PDE4) inhibitor that effectively modulates various inflammatory mediators, has demonstrated efficacy with a favorable safety profile in several RCTs involving PsA patients with peripheral involvement (138).

These trials excluded patients with TB disease, but they did not require TBI screening for enrolment. This finding is noteworthy: patients with TBI treated with apremilast without preventive therapy showed no instances of reactivation (139).

Analysis of pooled data from the PALACE I–II–III studies involving a total of 1493 PsA patients, reported zero cases of TB disease (140). A comprehensive retrospective analysis using a large US-based claims database, including patients diagnosed with psoriasis and/or PsA who were administered at least one dose of apremilast between 2014 and 2018, identified only two cases of TB disease among 10074 patients (141).

Overall, available data highlight the minimal association of apremilast with TB reactivation. Notably, the prescribing information for apremilast does not mention the necessity for TB screening before initiation of treatment.

### 4.8 JAK inhibitors

Janus kinase inhibitors (JAK-i) are non-receptor tyrosine kinases associated with the cytoplasmic domain of type I and II cytokine receptors, which are activated after the engagement by their cognate ligands. Once phosphorylated, they phosphorylate signal transducers and activators of transcription (STATs), which then induce gene activation essential for cellular functions like signaling, growth, and survival (142).

The JAK family comprises four cytoplasmic non-receptor tyrosine kinases: JAK1, JAK2, JAK3, and TYK2. JAK-i are categorized into two generations. The first generation includes small molecules like baricitinib (BAR) and tofacitinib (TOF), which act as non-selective inhibitors of JAKs. In contrast, second-generation drugs such as filgotinib (FLG) and upadacitinib (UPA) exhibit more selective inhibitory activity against JAK1 than other JAK (143, 144). UPA and TOF are approved for the treatment of RA, PsA, and AS, whereas FLG and BAR are approved for RA only.

It can be speculated that the broad immunosuppressive effects exerted by the mechanism of action of JAK inhibitors, particularly through the downregulation of IFN-γ, TNF-α, and IL-6, could disrupt critical host defenses, including macrophage activation, granuloma formation, and Mtb containment.

Notably, in a BALB/c mice model, TOF was shown to diminish the control of Mtb, leading to increased bacterial replication in the lungs during chronic paucibacillary TB. This model is designed to replicate latency in a manner analogous to human TBI (145). Screening for TBI is recommended in the SmPC of all JAK-i.

Data on TOF and TB were pooled from 7061 patients across the completed 2 phase I, 10 phase II, 6 phase III, 1 phase IIIb/IV index studies, and 2 open-label LTE studies (total exposure 22875PY). TB disease was reported in 36 (0.5%) patients, with an IR of 0.2/100PY. Pulmonary and non-pulmonary TB occurred in 17 and 19 patients respectively, with most cases occurring in geographical regions endemic to TB (146). Twenty-six cases of TB disease were identified from a post-marketing surveillance analysis of TOF, on a total of 5671 RA patients for an IR of 0.21/100PY, most of which were from regions with high background IR for TB (147). Finally, a retrospective, single-center analysis from Western India reported 4 TB cases on a total of 102 RA patients treated with TOF (148).

Regarding baricitinib, an integrated study from 9 RCTs conducted over 20 countries in patients with RA and one LTE study with a follow-up period of up to 7 years, showed a total IR for TB disease of 0.2/100 PY (15 out of 3770 patients; 14744 PY) (149, 150). The IR did not increase with prolonged exposure and the events occurred mainly in endemic countries.

Data on TB risk in patients receiving UPA are pooled from 12 clinical trials (SELECT program) on 3209 patients with RA (9079.1PY), 907 patients with PsA (1872.3PY) and 182 with AS (320.1PY), showed only one case of TB reactivation (151–153). The long-term extensions analysis on 3209 RA patients for 11661.5PY, recorded one case of disseminated TB, one case of peritoneal TB, two cases of pulmonary TB, one case of female genital tract TB and 174 cases of TBI reactivation (154). No cases of TB disease were recorded in the RCTs and LTE studies conducted in PsA and AS patients, whereas a total of 51 cases of TBI reactivation were reported (155–157). We still have a few real-life data on UPA. In two recent prospective longitudinal multicenter Italian studies in RA patients enrolling 71 and 60 patients respectively, neither TB disease nor new TBI were detected during the 6 months follow-up (158, 159).

The FINCH programme, a 52-week phase 3 RCT evaluated FLG in 833 RA patients, recorded no cases of TB disease (160).

Ninenty-one cases of TB disease reactivation and no new onset TB disease cases were reported in the DARWIN clinical trial and its LTE analysis on 739 RA patients (161). Overall, these findings suggest that JAK-i carry a risk of TB, particularly in endemic regions.

## 5 Screening strategies and preventive therapy for TB

It is estimated that approximately one-fourth of the global population has an immune response to Mtb, indicating previous exposure or infection with the bacterium (1). Since 2015, the WHO has recommended screening and treating TBI in populations at higher risk of progression to the disease, within preventive actions of the WHO End TB Strategy (3, 162).

Patients with autoimmune diseases candidates for biological treatment are considered at risk of progressing to TB. Therefore, International guidelines (163) recommend screening and TBI preventive treatment of those with a TBI diagnosis who are candidates for biological treatment. Patients are screened for TBI either using skin tests, or interferon-γ release assays (IGRAs) based on the national guidelines in place (6, 164). Skin tests are based on the intradermal inoculation in the forearm of the purified protein derivative (PPD) as in the tuberculin skin tests (TST), or on ESAT-6 and CFP-10, as in the new generation of skin tests (165).

IGRAs are blood tests based on *in vitro* stimulation with ESAT-6 and CFP-10; the read-out is based on IFN-γ or IP-10 detection that can be performed in automated or semi-automated ways (6, 164).

In countries with BCG vaccination, where TST may show false positives, ESAT-6 and CFP-10 based assays (IGRAs or skin tests) are preferred. If either the skin test or IGRA is positive, the patient is considered with TBI (162, 166) and will undergo a chest X-ray (CXR) to exclude TB disease (162). A baseline CXR can be useful also for those who score negative on skin tests or IGRA to evaluate lung apical scores compatible with spontaneously healed TB, such as non-calcified nodules with distinct margins and fibrotic linear opacity (167). If TBI is diagnosed from a positive skin test or IGRA without lung lesions, or based solely on lung apical scars, preventive treatment is offered to those at high risk to develop TB (162).

TB preventive therapy aims to eliminate the remaining replicating mycobacteria in the body, thus resulting in a lower risk of developing the disease. This has proven to be effective in preventing TB in several populations, including children (162, 168, 169).

## 6 TB preventive therapy drugs and drug regimens

TB preventive therapy comprises one or two antibiotics and is different from the therapy used for TB disease, in which four antibiotics are used to reduce the likelihood of acquired drug resistance. This assumes that in TBI the acquired drug resistance is unlikely, given the small number of viable bacteria present.

Drugs commonly used for TB preventive therapies may cause adverse reactions such as liver or neuro-toxicities (**Table 2**). Isoniazid (INH) is an oral antibiotic with intracellular and extracellular activity against Mtb. Isoniazid has been used globally, with an average protective effect for TB of 60% during the observation period (170). The duration is 6 months, although, in 1982, a randomized trial in subjects with fibrotic pulmonary lesions showed that the risk for developing TB disease compared with placebo was reduced by 21%, 65%, and 75%, respectively for 3-, 6-, or 9-months therapy, after 5 years of follow-up (171). However, a 6-month regimen was shown to be more cost-effective than 3 or 12 months for the reduction of side effects, regimen adherence and increased adherence (162, 172–174). Neuropathy can arise as an isoniazid side effect due to the inhibitory effect of isoniazid on the function of pyridoxine metabolites; therefore, pyridoxine (vitamin B6) supplementation is recommended especially in those with alcohol abuse, malnourished, and pregnant women (175).

**Table 2:** TB preventive therapies and their side effects.

Drug(s)	Dosage	Time	Major side effects	Pyridoxine supplementation
**INH**	5 mg/kg/die (max 300 mg/die)	6 months	Liver, peripheral neuropathy	Recommended
**INH**	5 mg/kg/die (max 300 mg/die)	9 months	Liver, peripheral neuropathy	Recommended
**Rifampicin**	10 mg/kg/die (max 600 mg/die)	4 months	Liver	
**INH+ Rifampicin**		3 months	Liver, peripheral neuropathy	Recommended
**Rifapentine+ INH**	Isoniazid 900 mg/weekly Rifapentine 900 mg/weekly	3 months	Liver, peripheral neuropathy, SDR*	Recommended
**Rifapentine+ INH**	Isoniazid 300 mg/day Rifapentine 600 mg/day	1 month	Liver, peripheral neuropathy	Recommended
**Levofloxacin**	<45 kg 750 mg/day; > 45 kg, 1g/day		Tenosynovitis, QT elongation, gastrointestinal symptoms	

SDR, systemic drug reaction, defined as either (1) hypotension, urticaria, angioedema, acute bronchospasm, or conjunctivitis; or (2) >4 flu-like symptoms, Age Impact on 3HP with >1 being grade 2 or higher.

The name of the drugs is highlighted in bold.

Rifamycins, a group of oral antibiotics such as rifampin and rifapentine, inhibit bacterial RNA synthesis by binding to the DNA-dependent RNA polymerase. These antibiotics are used to treat TBI by themselves or in combination with isoniazid to limit the side effects and the poor adherence to the long treatment duration of isoniazid (162).

Rifampicin regimens, like the 4-month course of rifampicin (4R) (162) or even shorter regimens, like the 3-month course of isoniazid and rifampicin (3HR), showed good safety and completion rates, particularly among children, with dispersible fixed-dose combinations aiding administration (176–178). For adults, however, liver toxicity and completion rate are comparable to those of longer isoniazid preventive therapy (177, 179). Administering a once-weekly dose of isoniazid and rifapentine for 12 weeks (3HP) is associated with lower rates of hepatotoxicity and higher completion rates when compared with isoniazid, although it was linked with the incidence of a hypersensitivity systemic immune response (177). Conversely, a recent meta-analysis indicated an increased incidence of grade 3 and 4 adverse events as well as a greater rate of treatment discontinuation for the 3HP regimen when compared with the 6–9 month isoniazid preventive therapy (IPT) (180).

A regimen of one-month daily INH and rifapentine (1HP) is an alternative for HIV-infected patients, and WHO conditionally recommended it for people aged above 13 years, although additional evaluations of safety and efficacy are needed in people without HIV (162).

For contacts of people with MDR-TB, WHO recommends using levofloxacin daily for six months to protect contacts following exposure to MDR-TB (181, 182).

For isoniazid and rifamycin therapies, as liver damage and neurotoxicity are the main side effects, conditions such as diabetes mellitus or alcoholism predisposing to neuropathy development, or chronic hepatitis B and C predisposing to liver injuries need to be carefully evaluated. Therefore, especially in patients with rheumatological disorders that often experience a metabolic syndrome (183), at baseline before starting therapy, we need to evaluate fast glycemia, glycated hemoglobin, HBsAg/Ab, hepatitis C virus Ab, and transaminase levels. To evaluate the risk of side effects to preventive therapies based on rifapentine, new strategies based on Whole-Blood Gene Signature have been proposed (184).

In patients treated with biological therapies, few studies are available regarding the side effects of preventive TB therapy (3, 185–187). A moderate and transient increase of isoniazid-induced liver damage has been reported (186, 188). Similarly, in a large Italian study, it was shown that 95% (280/295) of rheumatological patients completed TB preventive therapy with isoniazid and 96% with rifampicin (27/28). Importantly, patients who stopped taking isoniazid due to side effects successfully finished their treatment with rifampicin, showing that switching medications can still provide a good option for completing TB preventive therapy (3, 186, 187).

Although the data available are limited, this evidence suggests that patients undergoing biological therapy generally tolerate preventive treatments and complete the full course.

## 7 Management of preventive therapy in rheumatological patients

Before initiating preventive therapy, the physician needs to conduct a thorough medical history asking for previous exposure to TB cases and for previous liver disease, alcohol use, and concurrent treatments (to identify potential drug interactions). Patients should be informed about the symptoms of potential liver damage and should also be advised on whom to contact if these symptoms appear.

Blood control intervals of blood count, transaminases, γGT, and bilirubin should be fixed at initially 2-weekly then 4-weekly.

Methotrexate, commonly used for RA and PsA, carries a risk of liver toxicity (189). During TB treatment, it is crucial to balance the risk of arthritis flares with potential liver damage in patients taking methotrexate (190). Although specific guidelines are lacking, switching to less hepatotoxic csDMARDs, such as hydroxychloroquine or sulfasalazine, may be advisable, particularly for patients with stable disease activity. Patients with pre-existing liver conditions require even greater caution due to their increased susceptibility to toxic effects.

If preventive therapy is not tolerated, the rheumatologist should consider prescribing anti-rheumatic treatment with a low risk of TB disease reactivation. This procedure must be written and shared with the patient. Afterward, both the rheumatologist and the patient need to carefully monitor the occurrence of possible symptoms of mycobacterial reactivation to promptly isolate the patient to avoid further transmission, diagnosis, and treatment.

Importantly, after a fully completed therapy for TB disease or TBI, no further TB therapy needs to be given. It is assumed that preventive therapy kills all mycobacteria, and therefore no further preventive TB treatment is needed. IGRA results can remain positive, even after preventive therapy (191, 192), because these tests indicate the presence of an immune response against Mtb, not the presence of Mtb itself (193). Few data are present on the importance of repeated annual TB screening in a non-endemic area (185, 194). Evidence suggests that serial IGRA testing among low-risk patients on DMARDs results in a very low incidence of newly diagnosed TBI. Consequently, it is recommended to conduct targeted TBI screening based on risk factors related to TB —such as geographical origin, comorbidities like diabetes, or travel to endemic areas— before IGRA testing, rather than implementing universal annual screening in non-endemic regions (185, 194).

## 8 Conclusions

In conclusion, based on the available evidence, patients with chronic autoimmune arthritis under immunosuppressive treatment have an increased risk for TB reactivation. Among bDMARDs, TNF-α inhibitors are associated with an increased risk of TB progression compared to other treatments; however, the risk is not negligible, especially for JAK-i and anti-IL-6R agents.

Based on the WHO recommendations, either skin tests or IGRAs are acceptable for TBI screening. Stratification of TB risk is important to drive the bDMARDs choice. The preventive treatment for TB is well tolerated in patients undergoing b and tsDMARDs.
